# THF co-solvent pretreatment prevents lignin redeposition from interfering with enzymes yielding prolonged cellulase activity

**DOI:** 10.1186/s13068-021-01904-2

**Published:** 2021-03-09

**Authors:** Abhishek S. Patri, Ramya Mohan, Yunqiao Pu, Chang G. Yoo, Arthur J. Ragauskas, Rajeev Kumar, David Kisailus, Charles M. Cai, Charles E. Wyman

**Affiliations:** 1 Department of Chemical and Environmental Engineering, Bourns College of Engineering, University of California, Riverside, 900 University Ave, Riverside, CA 92521 USA; 2 BioEnergy Science Center (BESC), Oak Ridge National Laboratory (ORNL), Oak Ridge, TN 37831 USA; 3 Center for Environmental Research and Technology (CE-CERT), Bourns College of Engineering, University of California, Riverside, 1084 Columbia Ave, Riverside, CA 92507 USA; 4 Materials Science & Engineering Program, Bourns College of Engineering, University of California, Riverside, 900 University Ave, Riverside, CA 92521 USA; 5 Center for Bioenergy Innovation (CBI), Oak Ridge National Laboratory (ORNL), Oak Ridge, TN 37831 USA; 6 Joint Institute for Biological Sciences, Biosciences Division, Oak Ridge National Laboratory, Oak Ridge, TN 37831 USA; 7 Dept. of Paper and Bioprocess Engineering, College of Environmental Science and Forestry, State University of New York, Syracuse, NY USA; 8 Department of Chemical and Biomolecular Engineering, University of Tennessee Knoxville, Knoxville, TN USA

**Keywords:** Biomass, Pretreatment, Dilute acid, Tetrahydrofuran, Lignin, Enzyme, Cellulase, Protein, Scanning electron microscopy

## Abstract

**Background:**

Conventional aqueous dilute sulfuric acid (DSA) pretreatment of lignocellulosic biomass facilitates hemicellulose solubilization and can improve subsequent enzymatic digestibility of cellulose to fermentable glucose. However, much of the lignin after DSA pretreatment either remains intact within the cell wall or readily redeposits back onto the biomass surface. This redeposited lignin has been shown to reduce enzyme activity and contribute to rapid enzyme deactivation, thus, necessitating significantly higher enzyme loadings than deemed economical for biofuel production from biomass.

**Results:**

In this study, we demonstrate how detrimental lignin redeposition on biomass surface after pretreatment can be prevented by employing Co-solvent Enhanced Lignocellulosic Fractionation (CELF) pretreatment that uses THF–water co-solvents with dilute sulfuric acid to solubilize lignin and overcome limitations of DSA pretreatment. We first find that enzymatic hydrolysis of CELF-pretreated switchgrass can sustain a high enzyme activity over incubation periods as long as 5 weeks with enzyme doses as low as 2 mg protein/g glucan to achieve 90% yield to glucose. A modified Ninhydrin-based protein assay revealed that the free-enzyme concentration in the hydrolysate liquor, related to enzyme activity, remained unchanged over long hydrolysis times. DSA-pretreated switchgrass, by contrast, had a 40% drop in free enzymes in solution during incubation, providing evidence of enzyme deactivation. Furthermore, measurements of enzyme adsorption per gram of lignin suggested that CELF prevented lignin redeposition onto the biomass surface, and the little lignin left in the solids was mostly integral to the original lignin–carbohydrate complex (LCC). Scanning electron micrographs and NMR characterization of lignin supported this observation.

**Conclusions:**

Enzymatic hydrolysis of solids from CELF pretreatment of switchgrass at low enzyme loadings was sustained for considerably longer times and reached higher conversions than for DSA solids. Analysis of solids following pretreatment and enzymatic hydrolysis showed that prolonged cellulase activity could be attributed to the limited lignin redeposition on the biomass surface making more enzymes available for hydrolysis of more accessible glucan.

**Supplementary Information:**

The online version contains supplementary material available at 10.1186/s13068-021-01904-2.

## Background

Lignocellulosic biomass is a uniquely abundant resource for the sustainable production of non-petroleum derived fuels and chemicals [1]. Switchgrass, in particular, is being studied as a promising 2nd-generation feedstock for bioethanol production, due to its adaptability to varying climate conditions that would allow it to be grown on land not used for production of primary food or cash crops [2–4]. Pretreatment remains a key processing step aimed to improving the accessibility of the major cellulose fraction to enzymes that release glucose suitable for subsequent fuel–alcohol fermentations [5–7]. Pretreatment efficacy is generally governed by the reaction severity (temperature, duration, and acidity) that controls the extent of biomass deconstruction to sufficiently expose cellulose fibers from the complex lignocellulosic matrix. However, at elevated pretreatment severities, sugar degradation reactions reduce the total sugars available for subsequent hydrolysis [8]. Furthermore, sugar dehydration products such as furfurals can also inhibit fermentation causing a very complex optimization strategy for biomass pretreatment [9]. Several pretreatment technologies have been developed to disrupt the lignocellulose matrix and allow for greater accessibility to enzymes, thereby enhancing sugar yields during enzymatic hydrolysis [5]. However, achieving sufficiently high sugar yields during enzymatic hydrolysis still requires uneconomically high enzyme loadings, largely due to the presence of lignin which remains attached to the solid fraction after pretreatment [10, 11]. Lignin has been shown to competitively bind enzymes during enzymatic hydrolysis [12–14], thus, reducing the availability and activity of enzymes during hydrolysis and further affecting potential recovery and recycle of expensive enzymes [15]. Lignin and lignin-derived phenols have also been shown to inhibit cellulolytic enzyme activity during enzymatic hydrolysis [16, 17]. Therefore, an effective pretreatment should have high sugar yields during enzymatic hydrolysis at low enough enzyme loadings to reduce overall costs of producing 2nd-generation fuels from biomass.

Dilute sulfuric acid (DSA) pretreatment of lignocellulosic biomass has been shown to be effective at solubilizing the hemicellulose fraction while disrupting the lignocellulose matrix to allow for increased enzymatic access to carbohydrates [18, 19]. However, during DSA pretreatment, lignin has been shown to condense and relocate back on the surface, thus, acting as a physical barrier to enzymatic access of cellulose [20, 21]. Co-solvent Enhanced Lignocellulosic Fractionation (CELF) was recently developed as an advanced pretreatment technology capable of removing the majority of lignin from biomass, while realizing high sugar yields at low enzyme loadings [22]. The miscible mixture of tetrahydrofuran (THF) with water and dilute acid used for CELF has been demonstrated to preferentially solvate lignin, thus, allowing for its facile removal from cellulose and preventing lignin self-aggregation and redeposition [23, 24]. The resulting pretreated solids are highly digestible, achieving nearly theoretical glucose yields at a very low enzyme loadings [25, 26]. Although the substantial removal of hemicellulose and lignin has been shown to play a major role in higher glucan conversions for CELF-pretreated solids [24, 27], the role of residual lignin and its impact on enzyme activity on cellulase activity is not clearly understood. Therefore, the purpose of this study is to elucidate the mechanisms behind high enzyme activity during enzymatic hydrolysis of CELF-pretreated switchgrass by investigating the impact of lignin after pretreatment on enzymatic activity. First, enzymatic digestibility of DSA and CELF-pretreated Alamo switchgrass was measured over a range of enzyme loadings. In addition, the amount of enzyme adsorbed on the residual solids after solubilization of carbohydrates in DSA and CELF-pretreated switchgrass was measured to understand factors affecting sustained enzyme activity and sugar yields. Further, residual lignin fractions in pretreated solids after DSA and CELF were characterized using 2D HSQC NMR analysis. Finally, scanning electron microscope images were employed to picture the extent of surface morphology modifications of switchgrass samples by DSA and CELF pretreatments.

## Results and discussion

### Enzymatic digestibility of DSA and CELF-pretreated switchgrass at varying enzyme loadings

Alamo switchgrass was pretreated by DSA and CELF pretreatment using previously reported optimum reaction conditions that maximize sugar release following both pretreatment and subsequent enzymatic hydrolysis. The optimum pretreatment conditions for maximum sugar release are 160 °C, 20 min, and 0.5 wt% sulfuric acid for DSA and 150 °C, 25 min, and 0.5 wt% sulfuric acid for CELF [27]. The compositions of pretreated solids prepared at all pretreatment conditions were analyzed to determine the fate of components in the solids left by pretreatment. The mass of components in solids produced by application of the maximum sugar recovery pretreatment conditions for both DSA and CELF pretreatments were then adjusted to a basis of 100 g of unpretreated switchgrass (Additional file 1: Figure S1). As has been reported in previously published literature, the major difference between DSA and CELF-pretreated solids was the amount of lignin left in pretreated solids. DSA-pretreated solids contained 65% glucan, 4% xylan, and 32% acid-insoluble Klason lignin (K-lignin), whereas CELF-pretreated solids contained 86% glucan, 4% xylan, and 11% acid-insoluble Klason lignin (K-lignin).

**Fig. 1:**
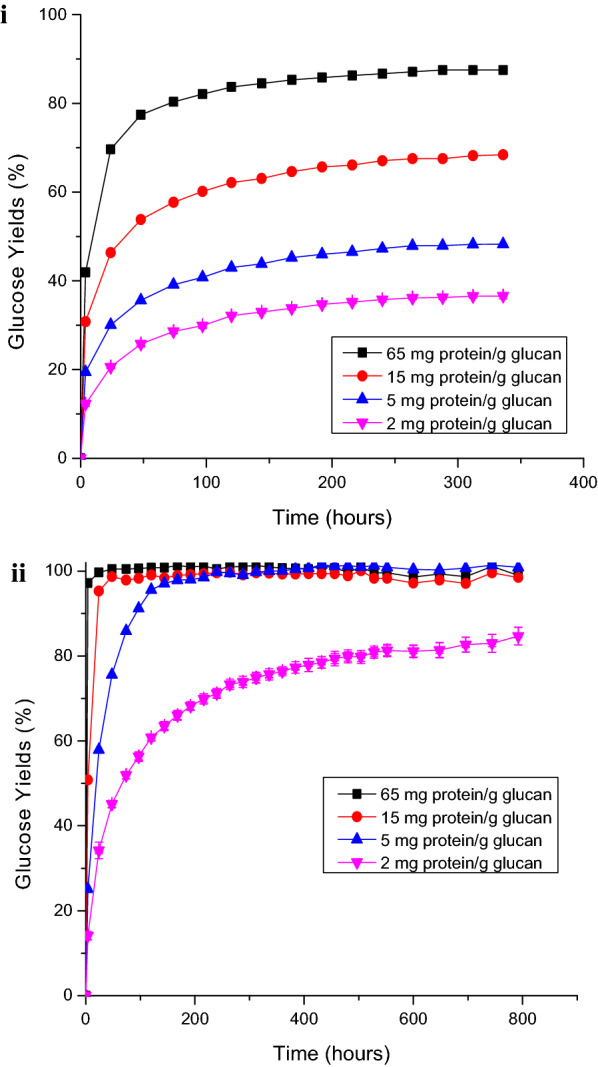
Comparison of glucose yields from enzymatic hydrolysis of solids prepared by **i** DSA and **ii** CELF pretreatments of switchgrass at cellulase loadings of 2–65 mg protein/g glucan in unpretreated switchgrass. Pretreatment reaction conditions were those that gave the highest total combined sugar yields at a loading of 65 mg protein/g enzyme, i.e., for DSA: 160 °C, 20 min, and 0.5 wt% sulfuric acid; for CELF: 150 °C, 25 min, and 0.5 wt% sulfuric acid at a 0.889:1 THF:water mass ratio

The digestibility of solids prepared by DSA and CELF pretreatments of switchgrass was determined for enzymatic hydrolysis at Accellerase^®^ 1500 cellulase loadings ranging from 2 to 65 mg protein/g glucan in unpretreated switchgrass. A glucan loading of 1 wt% was used during enzymatic hydrolysis to minimize the effect of product inhibition on cellulolytic enzymes and enable clear comparisons of lignin effects on enzyme activity. Enzyme loadings were based on unpretreated switchgrass so as not to penalize a pretreatment if it released more glucose in Stage 1. Ten days of hydrolysis at 65 mg protein/g glucan enzyme loading achieved a maximum glucose yield of 88% from DSA switchgrass (Fig. 1i). On the other hand, CELF-pretreated switchgrass reached 100% glucose yields in less than 2 days for both 65 and 15 mg/g enzyme loadings, and in 14 days at a 5 mg/g enzyme loading (Fig. 1ii). Further, at a considerably lower enzyme loading of just 2 mg/g, CELF-pretreated switchgrass continued to be enzymatically hydrolyzed for as long as 5 weeks while enzymatic hydrolysis of DSA-pretreated switchgrass virtually stopped after 2 weeks. This prolonged activity of cellulase enzymes on CELF-pretreated switchgrass could be attributed to the low lignin content of CELF-pretreated solids compared to DSA-pretreated solids in that lignin has been shown to unproductively bind cellulase as well as block the surface of cellulose substrate [21, 28, 29]. Thus, our results strongly suggest that the extent of lignin removal from biomass is critical to achieving high glucose yields during enzymatic hydrolysis particularly at low enzyme loadings. Further, the low amounts of lignin in pretreated solids could be responsible for the prolonged cellulose activity during hydrolysis.

### Enzyme-lignin binding during enzymatic hydrolysis of DSA and CELF switchgrass

**Fig. 2:**
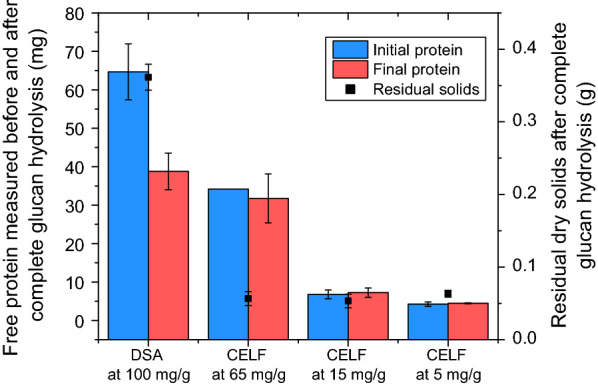
Initial and final protein measured in solution before (at 0 h) and after complete glucan removal by enzymatic hydrolysis at a 10 g/L glucan loading and cellulase loading of 100 mg protein/g glucan in unpretreated switchgrass for DSA-pretreated solids and 65, 15, and 5 mg protein/g glucan for CELF-pretreated switchgrass solids. Residual dry solids after complete glucan hydrolysis for DSA and CELF-pretreated switchgrass are shown on the right axis. Initial dry solids loading was 0.56 g for DSA and CELF pretreatments

Since CELF resulted in highly digestible solids and prolonged enzymatic activity, it was sought to understand mechanisms that could account for such enhanced enzyme performance through investigation of lignin fate after pretreatment. Although it was previously demonstrated that cellulose from CELF pretreatment had similar specific accessibility to cellulase enzymes [26], it appears that enzyme effectiveness (i.e., unit sugar produced/unit amount of bound enzyme) for CELF-pretreated biomass may be much higher than for DSA treated solids, owing to the lower lignin content of CELF solids. To assess this possibility. free protein concentration in the liquid was measured before (at 0 h) and after complete enzymatic hydrolysis of the glucan in solids produced by DSA and CELF pretreatments of switchgrass at 10 g/L glucan loadings, with the results shown in Fig. 2. Similar to what was shown in Fig. 1, a very high enzyme loading of 100 mg protein/g glucan was needed to achieve complete solubilization of glucan in DSA-pretreated switchgrass. On the other hand, for solids from CELF pretreatment of switchgrass, complete glucan solubilization was achieved at reduced enzyme loadings of 65, 15, and even at 5 mg protein/g glucan. The amount of initial and final protein in solution for DSA-pretreated solids revealed a 40% drop in free protein after enzymatic hydrolysis, whereas no significant drop in free protein amount was measured after hydrolysis of CELF-pretreated solids. These results suggested that the high level of delignification by CELF resulted in negligible binding of enzyme to residual solids and prolonged enzymatic activity during enzymatic hydrolysis. However, as also shown in Fig. 2, this result could stem from the very low amounts of residual solids left after complete removal of glucan by enzymatic hydrolysis of CELF solids compared to DSA solids, thus, making small differences in enzyme concentration beyond the sensitivity of the protein measurement assay. Recovery of CELF solids, previously shown to be composed of 11% K-lignin, was 10.7% after enzymatic hydrolysis, consistent with them being comprised of mostly lignin.

**Fig. 3:**
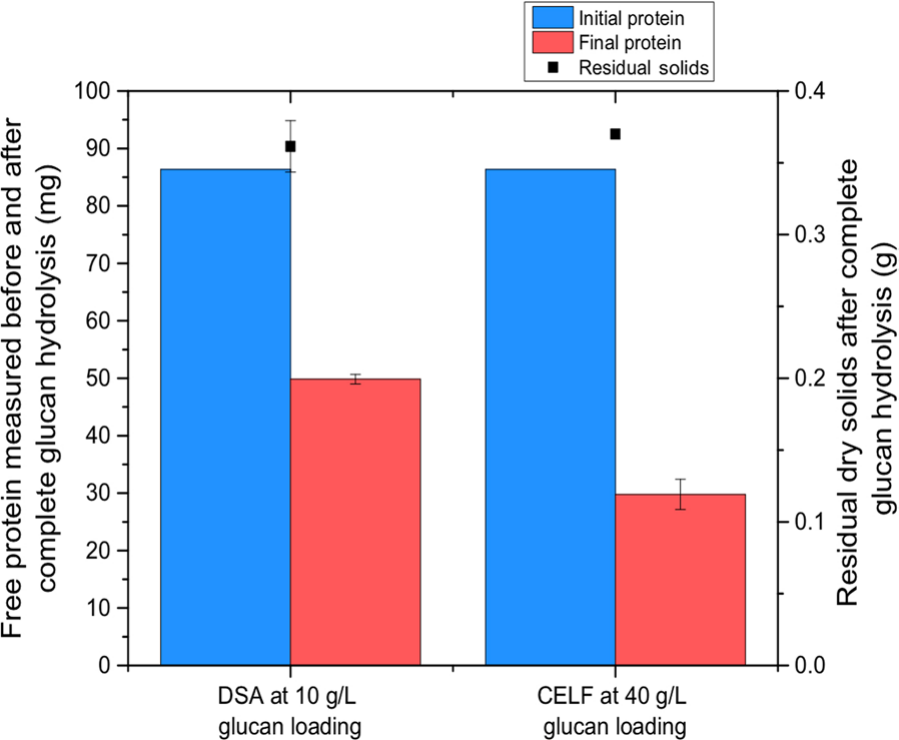
Initial and final free protein content (on the left axis) in enzymatic hydrolysis solutions measured before (at 0 h) and after complete glucan hydrolysis for DSA-pretreated switchgrass at 10 g/L glucan loading and CELF-pretreated switchgrass at 40 g/L glucan loading. Initial protein added in both cases was 1.72 g/L. Residual dry solids after complete glucan hydrolysis for DSA-pretreated switchgrass and CELF-pretreated switchgrass are shown on the right *y*-axis

Since Fig. 2 shows that residual solids after enzymatic hydrolysis of CELF-pretreated switchgrass, composed mostly of lignin, adsorbed negligible amounts of cellulase enzyme at equal glucan loadings, the question arises as to whether differences in DSA and CELF lignin could be responsible for this result. Therefore, enzyme adsorption by equivalent amounts of residual lignin was investigated to understand the enzyme binding behavior of lignin on solids prepared by DSA and CELF. The glucan loading for CELF solids was increased to 40 g/L to match the amount of lignin present in a 10 g/L glucan loading of DSA-pretreated switchgrass. The initial amount of enzyme was kept at 1.72 g protein/L for both cases to give an equivalent loading of 350 mg protein/g lignin. This approach was applied to ensure complete solubilization of both substrates and allow a direct comparison to the total amount of free protein after complete glucan solubilization. Figure 3 shows that after complete glucan saccharification, equal amounts of residual solids remained. However, because the final amount of free protein for CELF-pretreated solids was 65% less than the initial amount, this difference indicated that CELF residual solids containing mostly lignin actually adsorbed > 50% more enzyme on a per gram of lignin basis than residual DSA lignin.

A potential hypothesis to explain these differences is that lignin in pretreated solids prepared by aqueous pretreatments, such as DSA, are largely comprised of lignin globules deposited on the cellulose surface [20, 30] and this lignin may not adsorb as much enzyme as lignin in the lignin–carbohydrate complex (LCC). On the other hand, during CELF pretreatment, lignin solubility is maintained due to presence of the THF co-solvent, preventing redeposition onto cellulose [23]. Therefore, the lignin remaining in CELF-pretreated solids was mostly locked within the LCC, which in turn could bind more cellulase. On the other hand, because DSA-pretreated solids contained a very large amount of lignin redeposited on the cellulose in addition to LCC lignin, DSA lignin bound a lower mass of cellulose per gram of lignin.

**Fig. 4:**
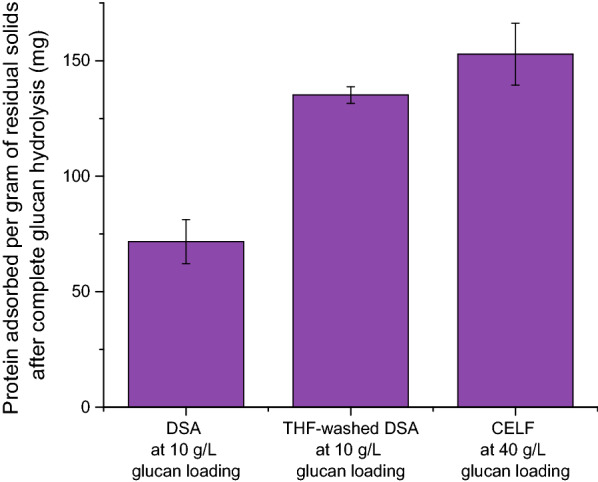
Protein adsorbed per gram of residual dry solids containing mostly K-lignin resulting from complete enzymatic hydrolysis of DSA and THF-washed DSA-pretreated switchgrass at 10 g/L glucan loading, and CELF-pretreated switchgrass at 40 g/L glucan loading. Initial protein added in all cases was 1.72 g/L

It has previously been suggested that redeposited lignin globules onto the cellulose surface do not strongly bind enzymes, but merely provide a physical obstacle between cellulose and enzymes during hydrolysis [21]. To test this hypothesis, lignin deposited on the cellulose surface of DSA-pretreated solids was removed by washing once with 500 mL THF at room temperature. Additional file 1: Figure S2 shows that THF washing removed surface deposited lignin without removing major carbohydrates or lignin from the LCC as demonstrated on unpretreated switchgrass and lignin-deposited Avicel (LDA). Bulk level compositional analysis of the THF-washed DSA switchgrass showed that 33% of the lignin was removed by the THF wash (Additional file 1: Figure S1). Conversely, THF washing of CELF-pretreated solids resulted in a negligible reduction in K-lignin content (Additional file 1: Figure S1). Following complete glucan solubilization of the THF-washed DSA solids, the amount of protein adsorbed per gram of residual solids was very similar to that of CELF residual solids (Fig. 4), suggesting that the majority of lignin in CELF-pretreated switchgrass is part of the LCC. This result also suggests that LCC lignin binds more cellulase than surface deposited lignin, possibly due to different structural and/or compositional differences.

### Structural characterization of lignin fractions from DSA and THF-washed DSA switchgrass

To study structural differences between redeposited lignin and lignin in the LCC, residual solids from DSA and THF-washed DSA switchgrass after complete carbohydrate digestion were characterized using 2D HSQC NMR. In addition, lignin in the wash liquid after THF washing of DSA switchgrass was recovered by evaporating THF to leave behind precipitated lignin and also characterized. Table 1 shows quantitative information regarding lignin subunits and inter-linkages. The data show that residual lignin in THF-washed DSA switchgrass contained a higher percentage of β-*O*-4 linkages and lignin recovered in the THF wash liquid contained a lower percentage of β-*O*-4 linkages when compared to lignin found in DSA-pretreated switchgrass. β-*O*-4 linkages are one of the major interunit linkages in unpretreated switchgrass [31]. These results provide further evidence that lignin in THF-washed DSA switchgrass (and likely, CELF switchgrass) is likely lignin remaining in the LCC, unlike the likely depolymerized and recondensed lignin recovered from the THF wash liquid, and thus, binds more enzyme during enzymatic hydrolysis. Since lignin present in DSA switchgrass is a combination of LCC lignin and recondensed lignin, the amount of bound enzyme per gram of lignin would be lower than that of lignin in THF-washed DSA switchgrass as the latter contained mostly LCC lignin.

**Table 1:** Quantitative information (expressed as %) for lignin subunits, hydroxycinnamates, and inter-linkages in DSA, THF-washed DSA switchgrass, and lignin recovered from wash liquid of THF-washed DSA switchgrass

	DSA switchgrass	THF-washed DSA switchgrass	Lignin recovered from wash liquid of THF-washed DSA switchgrass
Lignin subunits			
*S*	27.9	40.2	30.7
*G*	52.5	50.6	48.3
*H*	19.6	9.2	21.0
*S*/*G* ratio	0.53	0.79	0.64
Hydroxycinnamates			
FA	16.8	33.7	10.6
*p*CA	16.8	59.0	11.4
Interunit linkages			
β-*O*-4	18.4	40.2	9.7
β-5	6.1	8.0	4.8
β–β	0.6	1.2	0.5

Compositions are expressed as a fraction of *S* + *G* + *H*

*S*: syringyl unit, *G*: guaiacyl unit, *H*: *p*-hydroxyphenyl unit, FA: ferulate, *p*CA: *p*-coumarate, β-*O*-4: β-aryl ether, β-5: phenylcoumaran, β–β: resinol

**Fig. 5:**
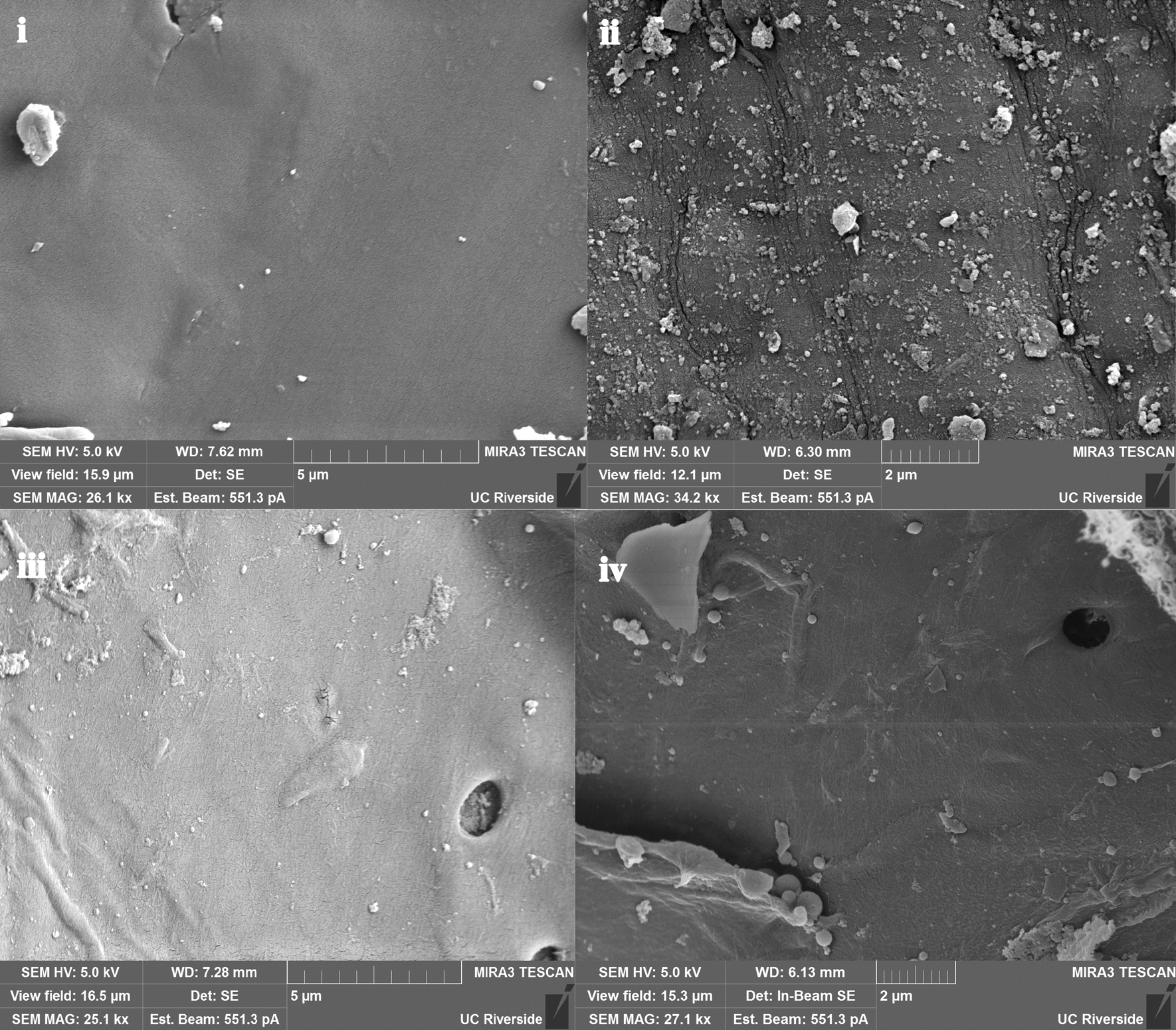
SEM images of switchgrass as **i** unpretreated, **ii** DSA pretreated, **iii** THF-washed DSA pretreated, and **iv** CELF-pretreated samples

Scanning electron micrographs were used to visualize differences in lignin deposited on the surface by DSA and CELF pretreatments of switchgrass. In addition, THF-washed DSA samples were imaged to examine the removal of surface deposited lignin by THF. After DSA pretreatment, a high concentration of lignin globules was observed on the surface of the pretreated biomass (Fig. 5ii), while washing with THF dramatically dropped the amount of lignin globules observable and the average diameter of individual droplets (Fig. 5iii). As expected, CELF-pretreated switchgrass appeared to have a low concentration of redeposited lignin globules (Fig. 5iv). These images confirmed that very little lignin redeposited back onto CELF-pretreated switchgrass and suggesting that majority of lignin in this material was still incorporated into the LCC, having little impact on enzyme activity.

CELF pretreatment was shown to delignify Alamo switchgrass to a very high extent, producing a glucan-rich solid that was highly digestible and resulted in prolonged hydrolytic enzymatic activity for at least 5 weeks, as compared to a plateau in enzymatic activity with DSA-pretreated switchgrass after 10 days of hydrolysis. These results motivated the further experiments to determine the reasons behind achieving prolonged enzymatic activity. The major bulk difference between CELF and DSA switchgrass was the significantly reduced amount of K-lignin present in CELF-pretreated switchgrass. Quantifying the amount of free protein in solution during enzymatic hydrolysis showed that the low amount of lignin in CELF-pretreated solids resulted in negligible amounts of enzyme being unproductively bound, thus, preserving enzymatic activity. DSA solids, on the other hand, adsorbed roughly 40% of the enzyme after complete glucan hydrolysis suggesting that the uncompetitive binding of enzymes to lignin was responsible for glucose yields plateauing after 10 days. Such a stark difference in enzyme binding between DSA and CELF lignin suggested that structural differences between lignin in the two pretreated solids could be the underlying reason for differences in enzyme binding properties. We believe that lignin in DSA switchgrass was a combination of native lignin in the LCC and lignin that was redeposited during pretreatment, whereas the small amount of lignin in CELF switchgrass was lignin in the LCC. THF washing of DSA switchgrass to remove redeposited lignin illustrated that the enzyme binding of LCC lignin is far greater than that of redeposited lignin (Fig. 4). Structural characterization of lignin in DSA switchgrass before and after THF removal of redeposited lignin as well as lignin recovered from THF wash liquid using 2D HSQC NMR and SEM images provided further evidence of the dual nature of lignin in DSA switchgrass, different from lignin in CELF switchgrass. In addition, the observation that THF washing removed a large fraction of surface redeposited lignin from DSA-pretreated solids while leaving most of the lignin on CELF solids further supported the hypothesis that CELF solids contained very little lignin on the surface and that most of the measured K-lignin was part of the LCC. This result implies that most lignin was removed directly from the LCC during CELF likely due to the ability of the co-solvent to dissociate lignin and render its aryl-ether bonds more susceptible to cleavage and the co-solvent keeping depolymerized lignin in solution, thus, mitigating its redeposition onto cellulose [24]. This mechanism of simultaneous lignin removal with minimal redeposition likely reduced the amount of enzyme that could unproductively adsorb during enzymatic hydrolysis and resulted in faster initial enzymatic rates because barriers to enzyme action on the surface of the glucan-rich solids were significantly reduced.

**Fig. 6:**
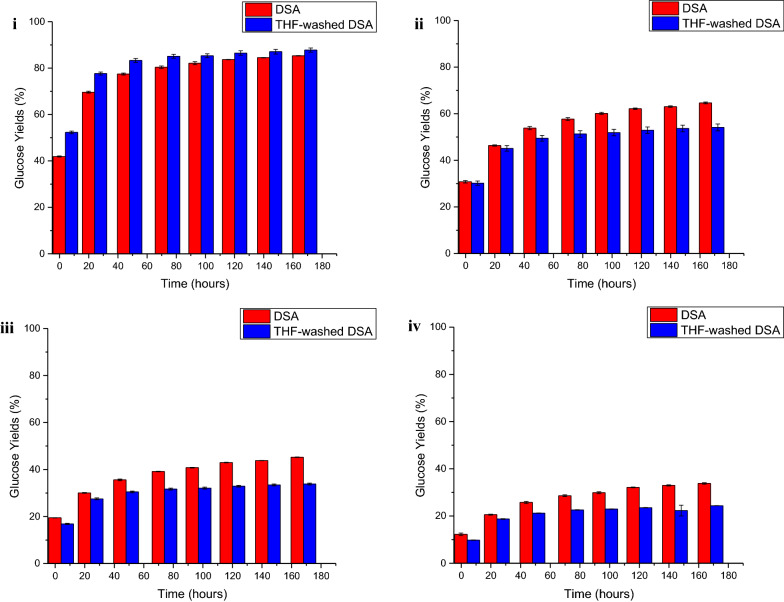
Comparison of glucose yields from enzymatic hydrolysis DSA and THF-washed DSA switchgrass at cellulase loadings of **i** 65 mg protein/g glucan, **ii** 15 mg protein/g glucan, **iii** 5 mg protein/g glucan, and **iv** 2 mg protein/g glucan in unpretreated switchgrass. Pretreatment reaction conditions for DSA: 160 °C, 20 min, 0.5 wt% sulfuric acid; THF wash performed with 500 mL of THF at room temperature

It must be noted, however, that removal of surface redeposited lignin alone did not enhance enzymatic yields [32, 33], particularly when significant lignin remained in the LCC. As shown in Fig. 6, enzymatic hydrolysis yields from THF-washed DSA switchgrass were only enhanced at the high enzyme loading of 65 mg protein/g glucan (Fig. 6i), while at lower enzyme loadings, glucose yields plateaued at lower values for THF-washed DSA solids compared to unwashed DSA solids (Fig. 6ii–iv). It is hypothesized that this difference was due to redeposited lignin shielding lignin in addition to cellulose in the LCC from enzymes. Thus, THF washing of redeposited lignin from the surface exposed considerably more of the remaining LCC lignin to enzymes that were in turn unproductively bound earlier in the hydrolysis process. When high enzyme loadings were applied to DSA solids, enough enzyme could be left in solution despite some binding to lignin in the LCC that cellulose could still be hydrolyzed to glucose with high yields. However, at lower enzyme loadings, significant amounts of enzyme may be bound to the exposed lignin in the LCC earlier, thus, resulting in lower glucose yields.

**Fig. 7:**
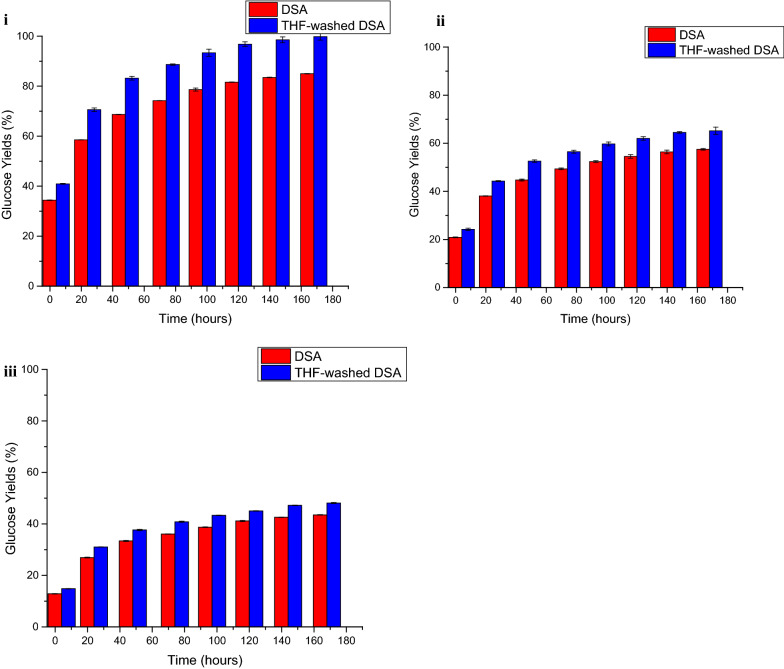
Comparison of glucose yields from enzymatic hydrolysis DSA and THF-washed DSA switchgrass with the addition of bovine serum albumin (BSA) at cellulase loadings of **i** 15 mg protein/g glucan, **ii** 5 mg protein/g glucan, and **iii** 2 mg protein/g glucan in unpretreated switchgrass. Pretreatment reaction conditions for DSA: 160 °C, 20 min, 0.5 wt% sulfuric acid; THF wash performed with 500 mL of THF at room temperature. BSA loading was 0.1 g BSA/g glucan

To support this hypothesis, bovine serum albumin (BSA) was added to bind with lignin in the LCC [12] prior to hydrolysis at the lower enzyme loadings. Upon doing so, the enhancement of glucose yields for THF-washed DSA switchgrass was greater than from DSA switchgrass (Fig. 7). This result suggested that the once lignin in the LCC was bound by BSA, the more exposed cellulose in THF-washed DSA switchgrass was more easily hydrolyzed than cellulose in DSA switchgrass. Faster hydrolysis rates were only observed with THF-washed DSA switchgrass once BSA was attached to lignin in the LCC to prevent it binding with cellulase. The lower hydrolysis rates and final yields observed without the addition of BSA supports the hypothesis that redeposited lignin in DSA switchgrass not only shields enzymes from cellulose but also from lignin in the LCC, thus, illustrating that removal of lignin from the LCC was crucial to minimizing enzyme binding to lignin and prolonging enzymatic activity.

CELF appeared to remove most of the lignin from the LCC, thus, leaving the bulk of the pretreated solid rich in cellulose. Even for application of low amounts of cellulolytic enzymes, the limited lignin redeposited on CELF solids implied that more of the cellulose in the CELF LCC was exposed to free enzymes, thus, enhancing enzyme–cellulose binding, as evidenced by the rapid initial rate of hydrolysis for CELF-pretreated switchgrass (Fig. 1). While these results suggest that although much less lignin is available to unproductively tie up enzymes, LCC lignin has a higher binding affinity for cellulolytic enzymes than surface redeposited lignin, further in-depth work is required to understand the differences in enzyme binding between these two types of lignin.

## Conclusions

THF as a co-solvent with water and dilute acid (CELF) can be an attractive pretreatment for biofuels production in that the highly digestible glucan-rich solids produced by CELF pretreatment can achieve nearly theoretical glucose yields at enzyme loadings as low as 5 mg protein/g glucan. CELF significantly enhanced lignin removal from switchgrass (up to 77% lignin removal) and substantially lowered lignin redeposition onto the cellulose surface compared to dilute acid alone. In addition, the preservation of cellulase activity for much longer periods of time during hydrolysis of CELF-pretreated solids compared to DSA highlights the importance of delignification of the plant cell walls prior to biological deconstruction. Further, the low lignin content of CELF-pretreated switchgrass was shown to result in much less cellulase being bound to lignin and thereby unavailable for further cellulose hydrolysis. This outcome contrasts with the 40% loss in cellulase to unproductive binding to lignin in solids produced by DSA, the current pretreatment benchmark [34]. The latter results in less cellulase available for hydrolysis in addition to reduced accessibility of cellulose to enzymes. On the other hand, although lignin left in CELF solids was mostly part of the LCC, CELF enhanced cellulase availability by dramatically reducing the total amount of K-lignin, thus, preserving enzymatic activity for prolonged hydrolysis times.

## Experimental section

### Materials

Senescent Alamo switchgrass provided by Genera Energy Inc. (Vonore, TN) was knife milled to ~ 1 mm particle size using a Wiley Mill (Model 4, Arthur H. Thomas Company, Philadelphia, PA) with a 1 mm particle size interior sieve. A fungal cellulolytic enzyme cocktail, Accellerase^®^ 1500, was provided by DuPont Industrial Biosciences (Palo Alto, CA). The protein concentration was measured, by applying the standard BCA method with bovine serum albumin as a standard, to be 82 mg/mL [35].

### Switchgrass pretreatment

Pretreatments were performed in a 1 L Hastelloy Parr^®^ autoclave reactor (236HC Series, Parr Instruments Co., Moline, IL) equipped with a double-stacked pitch blade impeller rotated at 200 rpm. For DSA reactions, solutions were loaded with 0.5 wt% (based on liquid mass) sulfuric acid (Ricca Chemical Company, Arlington, TX), while in CELF reactions, THF (> 99% purity, Fisher Scientific, Pittsburgh, PA) was added to a 0.5 wt% sulfuric acid solution in water at a 0.889:1 THF to acidic water mass ratio (or 1:1 by volume). Reaction conditions for CELF reactions were 150 °C for 25 min, while for DSA reactions were 160 °C for 30 min. These conditions were established based on previous sugar maximization studies for DSA and CELF pretreatment of Alamo switchgrass [27]. Prior to each pretreatment, milled switchgrass (7.5 wt%) was added to the solution and soaked overnight at 4 °C. All reactions were maintained at reaction temperature (± 1 °C) by convective heating with a 4 kW fluidized sand bath (Model SBL-2D, Techne, Princeton, NJ). The reaction temperature was directly measured using an in-line K-type thermocouple (Omega Engineering Inc., Stamford, Connecticut). Following pretreatment, solids were separated from the liquid by vacuum filtration at room temperature through glass fiber filter paper (Fisher Scientific, Pittsburgh, PA) and washed with room temperature deionized water until the filtrate was clear and pH reached neutral. The solids were carefully transferred to a ziplock bag and weighed. The moisture content of the solids was determined by a halogen moisture analyzer (Model HB43, Mettler Toledo, Columbus, OH). Lignin-deposited Avicel (LDA) was prepared as per Li et. al. [21].

### THF washing of switchgrass

Surface redeposited lignin was removed from the surface of pretreated switchgrass by washing the pretreated solids with THF. After the pretreatment hydrolyzate was separated from the pretreated solids, 500 mL of pure THF was used to soak the pretreated solids for 1 min after which, the liquid was filtered to produce THF-washed pretreated switchgrass. Lignin from the THF wash liquid was then collected by allowing the THF to evaporate in a chemical fume hood overnight. The resulting precipitated lignin was collected for characterization. THF washing of LDA was also performed as described above.

### Enzymatic hydrolysis

Enzymatic hydrolysis was performed as per the NREL protocol [36] in triplicate in 125-mL Erlenmeyer flasks with a 50 g total working mass made up of 50 mM sodium citrate buffer (pH 4.9) to maintain the hydrolysis pH and 0.02% sodium azide to prevent microbial contamination together with enough pretreated solids to result in approximately 1 wt% glucan. Accellerase^®^ 1500 cellulase loading was varied from 2 to 65 mg protein/g glucan in unpretreated biomass [37]. Bovine serum albumin (BSA) (Sigma-Aldrich Corp., St. Louis, MO) was added to select enzymatic hydrolysis flasks at a loading of 0.1 g/g glucan roughly 2 h prior to the addition of cellulase. Enzymatic hydrolysis flasks were placed in a Multitron orbital shaker (Infors HT, Laurel, MD) set at 150 rpm and 50 °C and allowed to equilibrate for 1 h before enzyme addition. Homogenous samples of approximately 500 μL were collected at 4 h, 24 h, and every 24 h and subsequently loaded into 2-mL centrifuge tubes (Fisher Scientific, Pittsburg, PA) and then centrifuged at 15,000 rpm for 10 min before analysis of the supernatant by HPLC. At the end of enzymatic hydrolysis, the residual solids were collected and bone dried at 65 °C.

### Biomass composition and sugar analysis

All chemical analyses were performed based on Laboratory Analytical Procedures (LAPs) documented by the National Renewable Energy Laboratory (NREL, Golden, CO). Compositional analysis of unpretreated and pretreated switchgrass was performed according to the NREL procedure (version 8-03-2012) in triplicates [38]. Liquid samples along with appropriate calibration standards were analyzed on HPLC (Waters Alliance e2695) equipped with a Bio-Rad Aminex^®^ HPX-87H column and RI detector (Waters 2414) with an eluent (5 mM sulfuric acid) flow rate at 0.6 mL/min. The chromatograms were integrated using an Empower^®^ 2 software package (Waters Co., Milford, MA).

### Quantification of free protein content in enzymatic hydrolysis liquid

A NaBH_4_-based modified Ninhydrin assay was used to quantify the protein in enzymatic hydrolysis liquor with reduced interferences from solubilized sugars [39]. In brief, 100 µL of sample or standard was incubated at room temperature for 60 min with 50 µL of 6.7 g/L NaBH_4_ in a 1.5-mL microcentrifuge tube. Bovine serum albumin (BSA) in the range of 0–2000 mg/L was used as the protein standard. This was followed by the addition of 300 µL of 9 M HCl and subsequent heating in a dry oven at 130 °C for 2 h. After cooling to room temperature, 100 µL of the sample was transferred into a fresh 1.5-mL microcentrifuge tube and neutralized with 100 µL of 5 M NaOH. Upon neutralization, 200 µL of 2% ninhydrin reagent (Sigma-Aldrich Corp., St. Louis, MO) was added to the tubes, which were then heated at 100 °C for 10 min in a dry oven. After cooling to room temperature, 500 µL of 50% (v/v) ethanol was added to each tube. Finally, 200 µL of colored solution was transferred to a 96-well microplate, and absorbance was read at 560 nm using a SpectraMax M2e Microplate Reader (Molecular Devices, Sunnyvale, CA). All samples were analyzed in triplicate.

### Scanning electron microscopy

Unpretreated and pretreated switchgrass samples were freeze-dried in a FreeZone 4.5-L Benchtop Freeze Dry System (Labconco, Kansas City, MO) for 24 h. Samples were sputter-coated with Pt/Pd (Cressington 108 Auto) for 90 s to form a conductive coating (~ 10–15 nm thickness), and subsequently examined with a Tescan MIRA3 GMU scanning electron microscope at an accelerating voltage of 5 kV and a working distance of 5 mm.

### Structural characterization of biomass residues after pretreatments

Prior to performing the 2D HSQC NMR analysis, each pretreated solid residues were ball-milled, and then hydrolyzed using C-Tec2 in 50 mM citrate buffer solution (pH 4.8) at 50 °C for 48 h. The recovered lignin-enriched residues were freeze-dried before dissolving in the NMR solvent (DMSO-*d*_6_). Two-dimensional ^1^H-^13^C heteronuclear single-quantum coherence (HSQC) spectra were collected using a Bruker standard pulse sequence (‘hsqcetgpsi2’). The central DMSO solvent peaks (*δ*_H_/*δ*_C_ = 2.49/39.5 ppm) were used for chemical shift calibration. Volume integration of cross peaks in HSQC spectra was carried out using Bruker’s TopSpin 3.5pl7 software. The THF extracted lignin was dissolved in DMSO and analyzed using HSQC without any purification.

### Calculations

Following HPLC quantification, the mass of each sugar was converted to the mass of the corresponding anhydrous form by multiplying cellobiose values by 0.95, glucose values by 0.90, and xylose values by 0.88 to compensate for the mass of water added during hydrolysis.

$$ {\text{Mass}}\;{\text{of}}\;{\text{sugar}}\;{\text{released}}\;{\text{in}}\;{\text{pretreatment}}\;{\text{hydrolysate}}\, = \,{\text{sugar}}\;{\text{concentration}}\;{\text{from}}\;{\text{HPLC}}*{\text{volume}}\;{\text{of}}\;{\text{pretreatment}}\;{\text{hydrolysate}}{.} $$

$$ {\text{Volume}}\;{\text{of}}\;{\text{pretreatment}}\;{\text{hydrolysate}},\;{\text{L}} = \left( {{\text{total}}\;{\text{reaction}}\;{\text{mass}}{-}\left( {{\text{mass}}\;{\text{of}}\;{\text{wet}}\;{\text{pretreated}}\;{\text{solids}}*{\text{moisture}}\;{\text{content}}} \right)} \right)/{\text{hydrolysate}}\;{\text{density}}{.} $$

$$ {\text{Enzyme}}\;{\text{loading}} = {\text{mg}}\;{\text{of}}\;{\text{protein}}\;{\text{per}}\;{\text{gram}}\;{\text{of}}\;{\text{glucan}}\;{\text{in}}\;{\text{enzymatic}}\;{\text{hydrolysis}}\;{\text{flask/glucan}}\;{\text{yield}}\;{\text{after}}\;{\text{pretreatment}}{.} $$

$$ \begin{aligned} & {\text{Enzymatic}}\;{\text{glucose}}\;{\text{yield }}\% \\ & = 100*\left( {{\text{Concentration}}\;{\text{of}}\;{\text{monomeric}}\;{\text{sugar}}\;{\text{measured}}\;{\text{by}}\;{\text{HPLC,}}\;{\text{g}}/{\text{L}}*{\text{anhydrous}}\;{\text{correction}}\;{\text{factor}}*{\text{total}}\;{\text{reaction}}\;{\text{volume}}\;{\text{of}}\;{\text{enzymatic}}\;{\text{hydrolysis}}\;{\text{flask}},\;{\text{L}}} \right)/{\text{Mass}}\;{\text{of}}\;{\text{glucan}}\;{\text{in}}\;{\text{enzymatic}}\;{\text{hydrolysis}}\;{\text{flask}}{.} \\ \end{aligned} $$

Following free protein quantification using a spectrophotometer, the free protein mass in solution was calculated as follows:

$$ {\text{Free}}\;{\text{protein}}\;{\text{measured}}\;{\text{in}}\;{\text{solution}},\;{\text{mg}} = {\text{free}}\;{\text{protein}}\;{\text{concentration}},\;{\text{mg}}/{\text{L}}*{\text{volume}}\;{\text{of}}\;{\text{enzymatic}}\;{\text{hydrolysis}}\;{\text{liquid}},\;{\text{L}}{.} $$

$$ \begin{aligned} & {\text{Protein}}\;{\text{adsorbed}}\;{\text{per}}\;{\text{gram}}\;{\text{of}}\;{\text{residual}}\;{\text{solids}},\;{\text{mg}}/{\text{g}}\;{\text{solids }} \\ & = \left( {{\text{free}}\;{\text{protein}}\;{\text{measured}}\;{\text{before}}\;{\text{hydrolysis}},\;{\text{mg}}{-}{\text{free}}\;{\text{protein}}\;{\text{measured}}\;{\text{after}}\;{\text{complete}}\;{\text{glucan}}\;{\text{hydrolysis}},\;{\text{mg}}} \right)/{\text{mass}}\;{\text{of}}\;{\text{residual}}\;{\text{solids}}\;{\text{after}}\;{\text{hydrolysis}},\;{\text{g}}{.} \\ \end{aligned} $$

$$ \begin{aligned} & \% {\text{Protein}}\;{\text{adsorbed}}\;{\text{after}}\;{\text{complete}}\;{\text{glucan}}\;{\text{hydrolysis }} \\ & = 100*\left( {{\text{free}}\;{\text{protein}}\;{\text{measured}}\;{\text{before}}\;{\text{hydrolysis}}{-}{\text{free}}\;{\text{protein}}\;{\text{measured}}\;{\text{after}}\;{\text{complete}}\;{\text{glucan}}\;{\text{hydrolysis}}} \right)/{\text{free}}\;{\text{protein}}\;{\text{measured}}\;{\text{before}}\;{\text{hydrolysis}}{.} \\ \end{aligned} $$

## Supplementary Information

**Additional file 1. Supplementary Information**

## Data Availability

The datasets supporting the conclusions of this article are included within the article and its additional files.
