# Supplementary material for: THF co-solvent pretreatment prevents lignin redeposition from interfering with enzymes yielding prolonged cellulase activity
Source: Biotechnol Biofuels. 2021 Mar 9;14:63. doi: 10.1186/s13068-021-01904-2 (PMC7944909; doi:10.1186/s13068-021-01904-2)
Supplement: Supplementary file 1 — Additional file 1. Supplementary Information [file 13068_2021_1904_MOESM1_ESM.docx]

**Additional file 1**

To study the effect of enzyme binding between lignin still linked to the LCC and lignin redeposited on the cellulose surface after dilute acid pretreatment, DSA switchgrass was washed with 500 mL of room temperature THF. The effect of the THF wash on removing redeposited lignin is shown in Figure S1. From bulk compositional analysis, roughly 33% of acid insoluble Klason lignin (K-lignin) was removed by the THF wash, indicating that one-third of K-lignin was that redeposited onto cellulose during DSA. THF washing of CELF switchgrass, on the other hand, removed a very small amount of lignin. The small amount of lignin that was removed from CELF solids was presumed to be lignin that had precipitated from the solution as some THF evaporated at the top of the Buchner funnel during solid-liquid separation of the CELF mixture.

**Figure S1.** Mass of glucan, xylan, and lignin left in the solids produced by DSA and CELF pretreatments at conditions optimized for recovery of highest overall glucan and xylan yields and for THF-washed DSA pretreated solids. The values shown were adjusted to a basis of the content of each component in 100 g of switchgrass before pretreatment. Reaction conditions: DSA: 160°C, 20 minutes, 0.5 wt% sulfuric acid. CELF: 150°C, 25 minutes, 0.5 wt% sulfuric acid, 0.889:1 THF/water mass ratio.

THF washing of biomass samples at room temperature was utilized to solubilize surface deposited lignin and remove it from the solid samples. To ensure that this process in fact only removed surface deposited lignin and not lignin or carbohydrates in the LCC, unpretreated switchgrass and lignin-deposited Avicel (LDA) were washed with THF as well. As shown in Figure S2, upon washing with THF, no change in major carbohydrates or lignin in unpretreated switchgrass was observed. The mass lost was attributed to the extractives present in switchgrass that are typically solubilized during pretreatment (*40*). Additionally, upon washing LDA with THF, the deposited lignin was removed from the solid fraction, leaving behind cellulose-rich Avicel.

**Figure S2.** Mass of glucan, xylan, and lignin in unpretreated and THF-washed unpretreated switchgrass, and lignin-deposited Avicel (LDA) and THF-washed LDA. THF wash was performed with 500 mL of THF at room temperature.
